# A historical review: Surgical management of massive irreparable rotator cuff tears

**DOI:** 10.1177/17585732251397826

**Published:** 2025-11-20

**Authors:** Andrew Kailin Zhou, Dave Osinachukwu Duru, Saroop Nandra, Salma Chaudhury

**Affiliations:** 1 2707 University of Warwick, Warwick, UK; 2 2708 Department of Trauma and Orthopaedics, University Hospital Coventry and Warwickshire, West Midlands, UK; 3 2152 University of Cambridge, Cambridge, UK; 4 Department of Trauma and Orthopaedics, Addenbrookes Major Trauma Unit, 2153 Cambridge University Hospitals, Cambridge, UK

**Keywords:** Historical, review, massive cuff tears, management

## Abstract

**Background:**

Massive irreparable rotator cuff tears (MIRCTs) represent a complex clinical challenge, resulting in pain, pseudoparalysis, and functional disability. Over the past century, their surgical management has undergone significant evolution, from early open repairs to sophisticated reconstructive and joint replacement strategies.

**Methods:**

A historical narrative review was conducted, informed by a systematic search of PubMed/MEDLINE, Embase, and landmark texts. Major surgical innovations–open repair, tendon transfers, debridement, superior capsular reconstruction (SCR), subacromial spacers, and reverse shoulder arthroplasty (RSA)–were examined in the context of their development and impact.

**Results:**

Open repairs offered limited success for chronic MIRCTs. Subacromial decompression and debridement provided symptom relief in low-demand patients. Tendon transfers and graft interpositions emerged as joint-preserving strategies in select cases. RSA transformed management by reliably restoring function in pseudoparalysis and cuff tear arthropathy. Contemporary techniques such as SCR and biologic augmentation aim to preserve native anatomy, but outcomes remain inconsistent.

**Discussion:**

MIRCT management has progressed from salvage repairs to a diverse toolkit of joint-preserving and reconstructive options. No single strategy is universally superior, underscoring the need for patient-specific approaches. Ongoing comparative trials and surgical consensus efforts are critical to optimising care.

## Introduction

Massive irreparable rotator cuff tears (MIRCTs) are full-thickness tears involving at least two rotator cuff tendons (or >5 cm in size) that cannot be mobilised for a tension-free repair.^1,2^ Such tears often result in pain, weakness, and pseudoparalysis of the shoulder, and chronic cases can lead to cuff tear arthropathy due to superior humeral head migration with degenerative joint changes.^3^ Historically, MIRCTs have posed a formidable challenge, and its surrounding terminology is yet to be conclusively defined. The Delphi consensus study by Schumaier et al. provided the first expert-derived, standardised definition: a massive rotator cuff tear is as retraction of tendon(s) to the glenoid rim, measured in either the coronal or axial plane, and/or ≥67% of the greater tuberosity exposed, measured in the sagittal plane, as assessed on MRI or intraoperatively.^4^ By contrast, ‘irreparable’ lacks formal consensus, as definitions vary widely. Common criteria include duration of over 6 months, retraction beyond 5 cm, Goutallier grade-3 fatty infiltration, and the rupture of two or more tendons.^5^

Over time, surgical management of MIRCTs has evolved from early open repairs to a spectrum of modern solutions including arthroscopic techniques, tendon transfers, spacers, reverse shoulder arthroplasty (RSA), and biologic augmentations (Table 1). This review chronicles the evolution of surgical management of MIRCTs from the earliest documented attempts to the present day, highlighting key technological advances (e.g., arthroscopy, implants, imaging) and landmark studies that shaped current treatment paradigms[Table table1-17585732251397826].

**Table 1. table1-17585732251397826:** Comparative summary of surgical options for massive irreparable rotator cuff tears.

Procedure	Indications	Typical benefits	Main limitations/complications	Highest-level of evidence
Open repair	Younger or acute tears with limited retraction	Anatomical restoration	High re-tear rate in chronic MIRCTs	II–III
Debridement ± Biceps Tenotomy/Tuberoplasty	Low-demand, elderly, irreparable tears	Pain relief, short rehab	No strength restoration	II
Tendon transfer (Latissimus/lower trapezius/pectoralis major)	Young, active, irreparable posterosuperior or subscapularis tears	Restores motion and external rotation	Technically demanding; donor morbidity	III–IV
Superior Capsular Reconstruction (SCR)	Irreparable posterosuperior tears, intact subscapularis	Restores stability, preserves anatomy	Expensive, technical, uncertain superiority to partial repair	II
Subacromial Balloon Spacer	Low-demand, pseudoparalysis without arthritis	Short op time, minimally invasive	No proven benefit vs debridement	I
Bursal Acromial Reconstruction (BAR)	Emerging biologic option for cuff-deficient shoulders	Restores superior restraint, increases AHD	Limited evidence; short follow-up	IV
Reverse Shoulder Arthroplasty (RSA)	Elderly, pseudoparalysis, cuff-tear arthropathy	Reliable pain relief, function restoration	Complications (instability, notching, infection); longevity uncertain in <65 years	II

AHD = acromiohumeral distance.

## Methods

A structured historical narrative review was performed, informed by a systematic search of PubMed/MEDLINE, Embase, and reference lists of landmark surgical texts. Search terms included: ‘massive rotator cuff tear,’ ‘irreparable,’ ‘history,’ ‘surgery,’ ‘technique,’ ‘tendon transfer,’ ‘arthroplasty,’ and ‘reconstruction.’ No date restrictions were applied. Publications were screened for relevance to the evolution of MIRCT surgical management, focusing on historical milestones, technical innovations, and outcome data across eras.

Given that much early evidence comprised descriptive case reports or technical notes, a systematic review was not feasible. Therefore, a narrative framework was used, following SANRA (Scale for the Assessment of Narrative Review Articles) guidelines to ensure clarity of rationale, comprehensive coverage, and critical synthesis of developments across eras.^6^

## Early surgical attempts and open repair techniques

**Figure 1.**
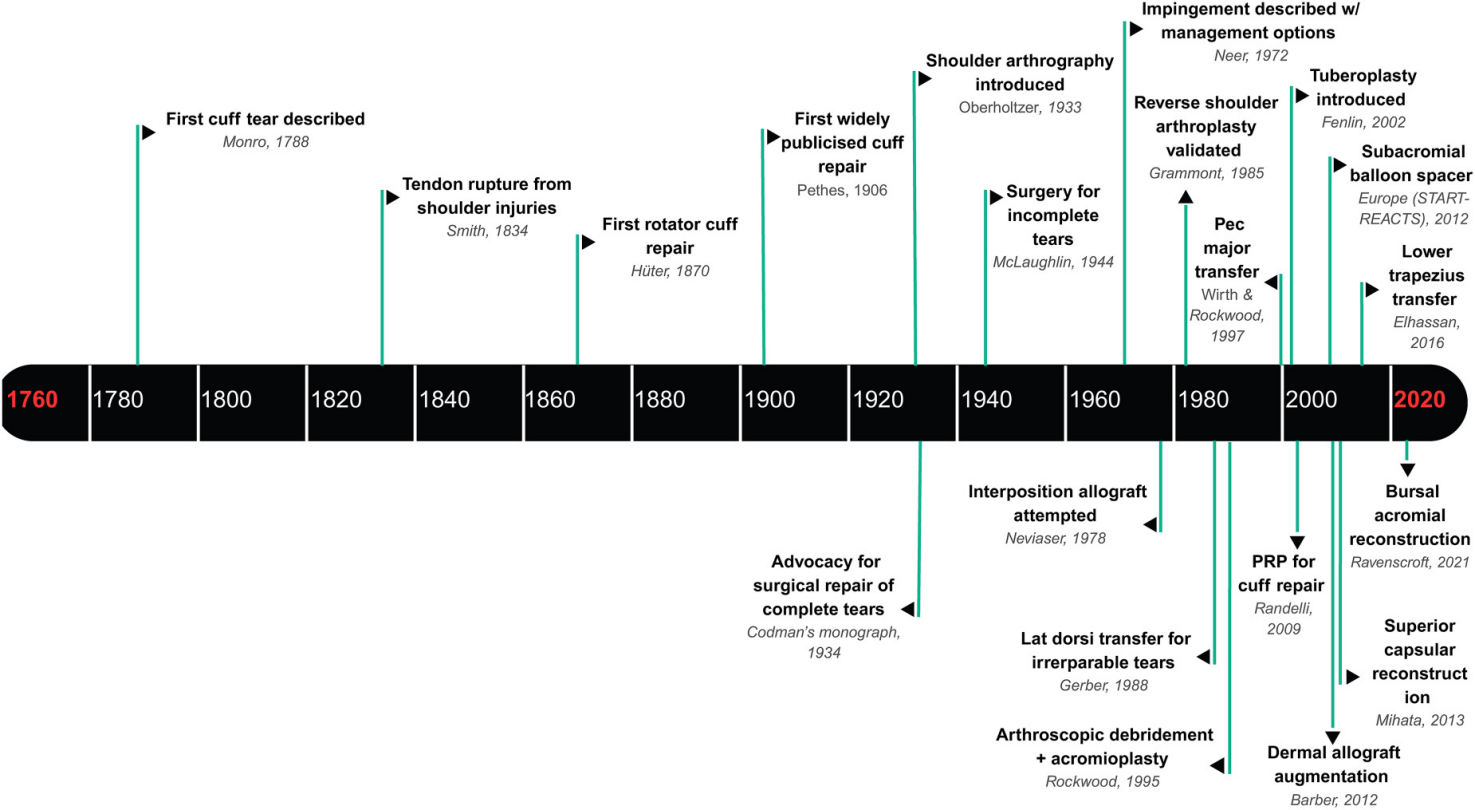
Historical timeline of major milestones in massive irreparable rotator cuff pathology and treatment. This timeline illustrates significant developments in the understanding and surgical management of rotator cuff injuries from the late eighteenth century to the early twenty-first century. Key events include the first description of a cuff tear by Monro in 1788, the first rotator cuff repair by Hüter in 1870, the introduction of shoulder arthrography in 1933, and Codman's seminal monograph in 1934 advocating surgical repair of complete tears. Advancements in surgical techniques such as arthroscopic procedures, tendon transfers, graft augmentations, and biologic approaches (e.g., PRP, dermal allografts) are marked across the timeline. The figure highlights the evolution of both diagnostic and therapeutic innovations culminating in contemporary strategies like superior capsular reconstruction and subacromial balloon spacers.

The recognition of rotator cuff tears dates back over two centuries. The first description and illustration of a rotator cuff tear are credited to Alexander Monro in 1788.^7^ In 1834, Smith described cases of tendon rupture after shoulder injury.^8^ The first surgical repair attempts followed decades later. German surgeon Karl Hüter reportedly performed a rotator cuff repair in 1870, although this was not widely publicised at the time^9^ (Figure 1). Many sources credit George Perthes for the first formal cuff repair in 1906.^7^ In the United States, Ernest Codman emphasised the importance of repairing torn rotator cuff tendons early; this may have been one of the first documented cuff repairs in 1909.^8^ Codman's 1934 monograph detailed 25 years of observations on rotator cuff pathology and advocated for surgical repair of complete tears.^8^ These early twentieth-century surgeons laid the foundation for rotator cuff surgery in an era predating modern imaging or anaesthesia, relying on open techniques and basic instrumentation. Such early surgical reports rarely differentiated between smaller and massive or irreparable tears, as imaging and intraoperative classification systems were not yet developed. Consequently, early outcomes reflected a heterogenous mix of tear severities.

By the mid-twentieth century, open rotator cuff repair had become more common, though outcomes for large chronic tears were poor. Surgeons recognised that massive, chronic tears often could not be directly reattached due to retraction and tissue degeneration. In the 1940s, McLaughlin described techniques for managing cuff tears, including debridement and partial repairs.^7,8^ Imaging advances began aiding diagnosis. Shoulder arthrography was introduced in 1933 by Oberholtzer (using air contrast) and refined with contrast dye by Lindblom in 1939, which helped identify partial versus massive tears radiographically.^10^ These early imaging techniques, alongside careful clinical exam, allowed surgeons to diagnose rotator cuff tears. However, treatment remained limited.^9^ Some surgeons attempted repair of the tissue, but reliable solutions were lacking, especially MIRCTs or significant retracted tears.^7,8^

## The era of decompression and debridement

In the 1970s, attention turned to the subacromial space as a source of impingement and pain in rotator cuff disease. Charles Neer in 1972 described the impingement syndrome and introduced anterior acromioplasty or subacromial decompression to relieve cuff attrition pain.^11^ While Neer's work focused on outlet impingement for reparable tears, the concept of decompressing the subacromial space was later applied to irreparable tears as a pain-relieving procedure. By removing bony spurs and the inflamed bursal tissue, surgeons aimed to allow the deltoid to glide unimpeded over a deficient cuff.

In 1995, Rockwood et al. formally described arthroscopic debridement with acromioplasty as a treatment for irreparable degenerative cuff lesions.^12^ In their classic series, 50 patients underwent subacromial decompression and rotator cuff debridement without repair; 83% experienced significant pain relief, and forward elevation improved from 105° to 140° on average.^12^ Rockwood emphasised that this was intended to reduce pain and restore shoulder function.^12^ This technique set a new treatment paradigm for low-demand or elderly patients with MIRCTs, simply clean out the joint and allow the patient to rehabilitate with an intact deltoid. Subsequent studies confirmed that debridement and biceps tenotomy (if the long head of biceps was pathologic) could yield pain relief and functional improvement in selected patients without the loss of bicep strength.^13^

Around the same period, tuberoplasty was introduced. In 2002, Fenlin et al. described reshaping the humeral greater tuberosity to create a smooth, congruent acromiohumeral articulation in the absence of a rotator cuff.^14^ By burring down the prominent tuberosity, the humeral head could articulate under the acromion without abrasive contact, could be considered as a ‘reverse subacromial decompression’.^14^ Early reports showed this technique improved pain in patients with massive tears.^15^ Tuberoplasty, often combined with debridement, provided another option to address pain in irreparable tears, especially for patients unsuited to complex reconstructions. While it did not restore normal mechanics, it prevented humeral head roughening of the acromion and mitigated superior migration in cuff-deficient shoulders.^16^

## Tendon transfer innovations

As surgeons grappled with restoring function in irreparable tears, tendon transfers emerged as a key strategy by the late twentieth century. The concept of rerouting a muscle-tendon unit to substitute for a deficient rotator cuff tendon has roots in earlier and brachial plexus injury surgeries, but its application to rotator cuff tears was pioneered in the 1980s.

The most influential was the latissimus dorsi tendon transfer for irreparable posterosuperior cuff tears. In 1988, Christian Gerber and colleagues reported transferring the latissimus dorsi tendon from its humeral shaft insertion to the superolateral humeral head to compensate for a deficient supraspinatus or infraspinatus tendon.^17^ Gerber's preliminary report in 1988 demonstrated that latissimus transfer could restore active elevation and external rotation in shoulders with massive tears that had an intact subscapularis.^17^ The original technique involved two incisions (axillary and deltoid-splitting approaches) to mobilise and reroute the tendon.^17^ Over the ensuing decades, latissimus transfer gained widespread adoption for younger, active patients with irreparable posterosuperior tears, with numerous series confirming durable improvements in motion and patient satisfaction.^18^

For irreparable subscapularis tears, the pectoralis major tendon transfer was developed. By the early 1990s, surgeons began transferring the sternal head of the pectoralis major to the lesser tuberosity to substitute for a deficient subscapularis. Wirth and Rockwood described this in 1997, and Gerber's group reported a series in 2003 confirming improved stability and internal rotation strength using pectoralis major transfers.^19,20^ Wirth and Rockwood published their technique where the superior 2.5–3 cm of the tendon is released from its insertion on the greater tubercle and transferred across the bicipital groove to the distal half of the greater tuberosity.^20^ Jost et al. showed that pectoralis major transfers can significantly reduce pain and restore shoulder function in patients with chronic subscapularis tears, especially when the posterior cuff is intact.^19^ While outcomes are somewhat variable (overhead use was not fully restored compared to baseline), this transfer remains a viable salvage for chronic subscapularis insufficiency in active patients who wish to avoid arthroplasty.^21^

Most recently, attention has turned to the lower trapezius transfer as an additional option for posterosuperior tears. Originally described by Elhassan et al. in 2016, the lower trapezius is transferred laterally, often with an Achilles tendon allograft extension, to recreate the line of pull of the infraspinatus.^22^ Early reports of arthroscopic-assisted lower trapezius transfers have shown promising improvements in external rotation and function for massive irreparable tears, particularly in patients who are younger or who failed latissimus transfer.^22^ The lower trapezius transfer is a joint-preserving alternative that avoids prosthetic replacement, and biomechanically it offers a more anatomic vector for external rotation than latissimus (since the trapezius originates more cranially).^22,23^ While follow-up is still limited, this technique underscores the ongoing innovation in tendon transfers to address irreparable cuff deficits.^22,24^

The field continues to advance with the emergence of arthroscopic approaches. In 2025, Lafosse et al. described the arthroscopic trans-scapular lower trapezius transfer for irreparable subscapularis tears, representing a major evolution in tendon transfer surgery.^25^ This technique allows a single-stage, minimally invasive reconstruction that reproduces the native subscapularis vector, optimises tensioning under direct visualisation, and avoids extensive deltoid detachment. Early clinical experience indicates that this modernised approach can restore internal rotation strength while maintaining external rotation function, highlighting how historical tendon-transfer principles continue to inform contemporary, arthroscopically enabled reconstructions.

## Joint replacement solution: RSA

For patients with massive cuff tears who also developed arthritis or pseudoparalysis, prosthetic replacement eventually became a game-changer.^26^ Traditional anatomic shoulder replacements fail when the rotator cuff is absent, because the prosthesis would destabilise without cuff support. Early attempts at constrained or fixed-fulcrum implants in the 1970s had high failure rates.^27^ The breakthrough came with Paul Grammont's development of reverse total shoulder arthroplasty (RSA) in the 1980s.^28^ Grammont's insight was to make the centre of rotation of the shoulder more medial and distal, effectively increasing the deltoid's lever arm in the absence of a rotator cuff.^28^ By reversing the ball-and-socket and using a strictly non-anatomic geometry, the deltoid muscle could elevate the arm even with a deficient cuff.^27^

In 1985, Grammont and colleagues validated this reverse prosthesis concept in the lab and implanted the first prototype with a cemented glenosphere.^28^ Over the next decade, they refined the design into the Grammont-style RSA (the Delta prosthesis), which demonstrated for the first time that patients with cuff tear arthropathy could regain overhead function and pain relief reliably.^27^ RSA was revolutionary: studies in the early 2000s by Boileau, Frankle and others showed that RSA could improve active forward elevation from 40–60° pre-op to well over 100° post-op in cuff tear arthropathy patients, with significant pain reduction and significant improvement in patient reported outcome measures, albeit with some complications (notching, hardware issues, and decreased ROM in the longterm).^29,30^ To address these concerns, the centre of the glenosphere was lateralised in subsequent iterations of RSA.^30^ Lateralisation restores deltoid wrapping, tensions the residual rotator cuff and external rotators, reduces scapular notching, and improves rotational strength.^31,32^ Lateralised designs broadened the indications to younger more active and revision populations.^33^

By 2004, the RSA was approved in the United States, and it quickly became the treatment of choice for elderly patients with massive irreparable tears, especially when accompanied by glenohumeral arthritis or profound pseudoparalysis; it has been highly successful.

In the United Kingdom, RSA has become the predominant treatment for cuff-deficient shoulders. Since the commencement of the National Joint Registry (NJR) in 2012, the percentage of shoulder arthroplasties that are RSAs has increased from 25.7% to 70.4% in 2024.^34^

The success of RSA has significantly influenced treatment algorithms; many surgeons will proceed straight to RSA for an irreparable tear in an older adult rather than attempt tendon transfers or other complex repairs.

## Interposition grafts, SCR, and bursal acromial reconstruction

Another major avenue of innovation has been the use of grafts to bridge or reconstruct the rotator cuff tear gap. The idea of placing a tissue patch to span an irreparable rotator cuff defect arose decades ago. In 1978, Neviaser et al. first attempted an interposition graft, using a freeze-dried rotator cuff allograft to fill a chronic massive tear.^35^ This novel technique had disappointing results as 14 of 16 patients had poor outcomes.^35^ Similarly, Nasca in 1988 reported only two satisfactory results out of 13 cases using an allograft patch.^36^ Such early studies dampened enthusiasm, as they suggested that simply bridging the gap with cadaver tendon did not reliably restore function. Over the next two decades, however, graft materials and techniques improved. Moore et al. found that human allograft patches often failed radiographically, and Sclamberg et al. showed a porcine small-intestine submucosa patch re-tore in 10 of 11 cases.^37,38^ On the other hand, some successes were noted. Shepherd et al. reported long-term (9.7 years) follow-up of a Gore-Tex (PTFE) synthetic patch in 5 patients with 80% of repairs still intact and improved motion.^39^ Seker et al. likewise found 91% of PTFE patches intact at 2 years with strength gains.^40^ In Europe, synthetic mesh interposition (Mersilene tape or Dacron grafts) showed initial functional improvement, though very long-term results revealed retears and no prevention of arthropathy in many cases.^41,42^ The mixed results of these efforts taught surgeons that grafts could *provide* mechanical bridging and perhaps a biologic scaffold, but material choice and technique were critical.

A true breakthrough in graft utilisation came from Japan in 2012 and 2013: SCR. Mihata and colleagues conceived SCR to restore the stability of the glenohumeral joint by reconstructing the superior capsule (rather than the tendon itself) using a graft. Mihata's technique involves fixing a thick fascial graft (initially autologous fascia lata) from the superior glenoid to the greater tuberosity, spanning the gap where the supraspinatus would normally be.^34,35^ The SCR is not a direct tendon repair, but by re-establishing the superior capsule, it prevents the humeral head from migrating upward and allows the remaining intact cuff and deltoid to function more effectively. Mihata's 2013 study showed remarkably improved shoulder function and acromiohumeral distance restoration in patients with irreparable tears treated with SCR, with mid-term follow-ups showing maintained improvements and high rates of graft integrity.^43^ This sparked global interest, and SCR was rapidly adopted by surgeons in North America and Europe, often using acellular dermal allograft instead of fascia lata autograft. Early Western series also reported promising outcomes, though not all results were uniformly excellent.^44–46^

Notably, a recent comparative analysis has questioned whether SCR truly outperforms simpler treatments. A 2025 meta-analysis compared SCR to partial repair and found no statistically significant differences in functional outcomes between the two techniques.^47^ This suggests that while SCR can reliably improve stability, its clinical benefit over well-done partial repairs may not be dramatic in the short term. Additionally, SCR is technically demanding and costly due to graft expense. Still, it represents a major conceptual advance and continues to evolve (e.g., SCR with internal brace or use of biceps tendon as graft).^48,49^ The SCR embodies the trend of using biologic constructs to restore joint kinematics without outright replacing the joint.

Bursal Acromial Reconstruction (BAR) has recently emerged as a biologic interpositional technique for MIRCTs. The procedure involves fixation of a human dermal allograft or fascia lata autograft to the undersurface of the acromion to recreate a superior restraint analogous to the native subacromial bursa.^50^ The graft acts as a spacer, reversing superior humeral migration, increasing acromiohumeral distance, and reducing subacromial contact pressure. Seo et al. (2024) reported in a case series of 18 patients undergoing combined BAR and biologic tuberoplasty, acromiohumeral distance improved from 4.3 ± 4.1 mm pre-operatively to 9.2 ± 1.9 mm post-operatively (*p* < .001), with no graft failures at one year and significant improvement in function and pain scores.^51^ However, clinical evidence remains sparse, and there is currently no consensus on indications, graft type, thickness, or fixation method.^52^

## Subacromial balloon spacers

Among the more novel device-based treatments for MIRCTs is the subacromial balloon spacer**.** First introduced in Europe in 2012 (and commercially known as the InSpace balloon introduced by Stryker), this biodegradable balloon is arthroscopically inserted into the subacromial space and inflated with saline to act as a temporary cushion between the acromion and humeral head. The spacer maintains a separation that centres the humeral head, reducing superior migration and allowing the deltoid to elevate the arm more efficiently despite the torn cuff. The allure of the balloon spacer is its simplicity, a short surgical time, minimal invasiveness, and no need for tendon grafts or osteotomies.

To rigorously evaluate the balloon, a multicentre randomised controlled trial was conducted in the United States. Verma et al. compared the InSpace balloon versus arthroscopic partial cuff repair in patients with irreparable tears and intact subscapularis.^53^ The findings indicated that the balloon was not inferior to partial repair**.** At one-year follow-up, both groups had similar improvements in pain and function.^53^ However, it did not clearly surpass partial repair, and longer-term results are still needed (since the balloon dissolves, some have questioned whether benefits persist beyond a year). The START:REACTS trial is a landmarked multicentre, double-blind, randomised controlled study.^54^ Metcalfe et al. showed that the InSpace balloon does not outperform arthroscopic debridement and biceps tenotomy alone in functional outcomes and may even result in worse shoulder scores (Oxford Shoulder Score: balloon group: 30.3 ± 10.9 and debridement-only group: 34.3 ± 11.1 [95% CI −8.2 to −0.26], *p* = .037).^54^ The 24-month follow-up confirmed that no significant functional advantage emerged for the balloon group over time. These findings have led to a significant reassessment of the clinical role of the InSpace balloon, which is now used more selectively.

## Biologic therapies and augmentations

As our understanding of tendon biology has advanced, so too have efforts to augment rotator cuff healing with biologics. Massive cuff tears, especially chronic ones, often have poor healing capacity due to muscle atrophy and tendon degeneration. Modern research has investigated whether biologic adjuvants could improve healing in repairs or even non-operatively. One avenue has been platelet-rich plasma (PRP) and other growth factor concentrate. The hypothesis is that delivering a high concentration of growth factors to the repair site may stimulate cell proliferation and matrix formation.^55^ The application of PRP as an adjunct in arthroscopic rotator cuff repair was first introduced in 2009 by Randelli and colleagues in a pilot study that led to a randomised controlled trial.^56^ At 2-year follow-up, 12 patients experienced massive rotator cuff tears. PRP-treated patients demonstrated significantly improved outcomes at 3 months postoperatively, including greater external rotation strength (3.0 kg vs. 2.1 kg, *p* <.05), UCLA scores (26.9 vs. 24.2, *p* < .05), and Constant scores (65 vs. 57.8, *p* < .05). These benefits were not sustained beyond 6 months, and MRI-assessed tendon healing rates remained comparable (*p* = .88). To date, no study has specifically focused on the use of PRP as an adjunct in MIRCTs. However, PRP has been used to augment partial repairs or grafts. For instance, surgeons sometimes soak allograft patches in PRP or bone marrow concentrate before implanting them, hoping to enhance graft incorporation.^57,58^ The clinical impact of this practice is still unproven, and inconsistent results mean PRP is not standard for MIRCTs currently.

Perhaps the most practical biologic innovation in recent years for massive tears has been the refined use of dermal allograft patches. Acellular human dermal matrix provides a collagen-rich scaffold that can both bridge a defect and potentially facilitate host tissue infiltration and healing.^46^ Unlike the earlier synthetic patches, dermal allografts are biocompatible and have shown encouraging results as augmentation for large repairs. For example, a comparative study by Barber et al. found that augmenting a cuff repair with a dermal allograft resulted in higher healing rates compared to repair alone in cases of massive tears.^59^ However, the patient-reported outcome measures, although higher in the dermal allograft augmentation group, were not statistically different.^59^ In the context of irreparable tears, dermal allograft can be used either as an interposition (bridging) graft or as part of an SCR. The consensus is that biologic patches can provide some additional strength and possibly a healing boost, but technique is key, is good fixation, must be secure, and tension minimised.

## Strengths and limitations

This review draws together over a century of surgical innovation in the management of MIRCTs. A strength is the breadth of historical synthesis, capturing the evolution of surgical philosophy from early open repairs to contemporary arthroscopic and reconstructive techniques. However, several limitations must be acknowledged. Many early reports were not available in indexed databases and were accessed through manual reference searching; therefore, some historical papers may have been missed. The inclusion of studies across multiple decades introduces selection and publication bias, as older literature predominantly reports successful outcomes and lacks uniform reporting of complications. Furthermore, changes in MIRCT definitions over time create heterogeneity in patient populations, techniques, and outcome measures, limiting direct comparison. Survivorship bias may also be present, as procedures with poor results were less likely to be repeatedly published or cited. Finally, only English-language studies were reviewed, introducing language bias and potentially underrepresenting early non-English European and Asian contributions. These limitations are inherent to historical narrative reviews but were mitigated through transparent sourcing, triangulation of primary references, and use of contemporary framework definitions.

## Future research

Ongoing controversies in MIRCT management highlight the need for comparative, high-quality evidence across surgical strategies. The long-term durability of RSA in patients under 65 years remains uncertain, and its expanding indications require careful outcome tracking. Meanwhile, recent meta-analyses suggest no clear superiority of SCR over partial repair, yet potential differences in re-tear rates and cost-effectiveness warrant further study. Similarly, the clinical role of subacromial balloon spacers is being re-evaluated following high-level RCTs showing no advantage over debridement alone. Modern tendon transfers and biologic augmentation techniques continue to evolve toward arthroscopic and patient-specific approaches, yet optimal patient selection criteria, comparative efficacy between different transfer types, the role of combined procedures, and long-term outcomes remain areas requiring prospective comparative evaluation.

Equally important is the inclusion of patient perspectives in outcomes research to better understand functional expectations and quality-of-life impacts across different treatment pathways. Furthermore, a Delphi consensus process or national or international survey could provide valuable insight into current surgical practice patterns, including decision-making thresholds, the use of joint-preserving strategies, and intraoperative crossover or fallback procedures. These research efforts would help establish evidence-based standards and foster consensus among surgeons in the increasingly diverse landscape of MIRCT management.

## Conclusion

The management of MIRCTs has evolved over more than a century from empiric, surgeon-led repairs to a nuanced, algorithmic, and patient-centred discipline. Early open repairs and debridements reflected an era of surgical experimentation in which treatment choice was dictated largely by available technique rather than by patient characteristics. With improved imaging, classification systems, and biomechanical understanding, surgeons recognised that tear patterns, tissue quality, and muscle degeneration are profoundly heterogeneous across patients. This biological variability, combined with differences in age, functional demands, and expectations, necessitates individualised solutions rather than a single ‘best’ operation. This paradigm shift, from ‘what can be repaired’ to ‘what best serves the patient’, has been reinforced by modern outcomes research. Contemporary evidence demonstrates that while RSA can restore elevation and relieve pain, younger or high-demand patients often report lower satisfaction and functional recovery. Conversely, joint-preserving procedures such as partial repair, tendon transfer, or biologic reconstruction may better align with the priorities of maintaining strength, delaying prosthetic replacement, and preserving anatomy. As patient-reported outcome measures (PROMs) and return-to-activity data have become central to evaluating success, the field has increasingly embraced shared decision-making – balancing patient age, function, occupation, and expectations against anatomical constraints. The contemporary algorithm for MIRCT management therefore reflects the cumulative lessons of a century of innovation: that effective care requires integration of anatomical realism with patient-defined goals. The future of MIRCT surgery lies not in a single superior procedure, but in refining personalised pathways that optimise quality of life, function, and satisfaction across diverse patient populations.
